# ZEB2 Mediates Multiple Pathways Regulating Cell Proliferation, Migration, Invasion, and Apoptosis in Glioma

**DOI:** 10.1371/journal.pone.0038842

**Published:** 2012-06-26

**Authors:** Songtao Qi, Ye Song, Yuping Peng, Hao Wang, Hao Long, Xiaoli Yu, Zhiyong Li, Luxiong Fang, Aibing Wu, Weiren Luo, Yan Zhen, Ying Zhou, Yan Chen, Chunping Mai, Zhen Liu, Weiyi Fang

**Affiliations:** 1 Department of Neurosurgery, Nanfang Hospital, Southern Medical University, Guangzhou, People’s Republic of China; 2 Cancer Research Institute of Southern Medical University, Guangzhou, People’s Republic of China; 3 Department of Pathology, Basic School of Guangzhou Medical College, Guangzhou, People’s Republic of China; Wayne State University School of Medicine, United States of America

## Abstract

**Background:**

The aim of the present study was to analyze the expression of Zinc finger E-box Binding homeobox 2 (ZEB2) in glioma and to explore the molecular mechanisms of ZEB2 that regulate cell proliferation, migration, invasion, and apoptosis.

**Methodology/Principal Findings:**

Expression of ZEB2 in 90 clinicopathologically characterized glioma patients was analyzed by immunohistochemistry. Furthermore, siRNA targeting ZEB2 was transfected into U251 and U87 glioma cell lines *in vitro* and proliferation, migration, invasion, and apoptosis were examined separately by MTT assay, Transwell chamber assay, flow cytometry, and western blot.

**Results:**

The expression level of ZEB2 protein was significantly increased in glioma tissues compared to normal brain tissues (*P*<0.001). In addition, high levels of ZEB2 protein were positively correlated with pathology grade classification (*P* = 0.024) of glioma patients. Knockdown of ZEB2 by siRNA suppressed cell proliferation, migration and invasion, as well as induced cell apoptosis in glioma cells. Furthermore, ZEB2 downregulation was accompanied by decreased expression of CDK4/6, Cyclin D1, Cyclin E, E2F1, and c-myc, while p15 and p21 were upregulated. Lowered expression of ZEB2 enhanced E-cadherin levels but also inhibited β-Catenin, Vimentin, N-cadherin, and Snail expression. Several apoptosis-related regulators such as Caspase-3, Caspase-6, Caspase-9, and Cleaved-PARP were activated while PARP was inhibited after ZEB2 siRNA treatment.

**Conclusion:**

Overexpression of ZEB2 is an unfavorable factor that may facilitate glioma progression. Knockdown ZEB2 expression by siRNA suppressed cell proliferation, migration, invasion and promoted cell apoptosis in glioma cells.

## Introduction

Despite the fact that glioma is one of the most common primary brain tumors [1], surgery followed by chemotherapy remain the standard treatment [2], [3]. Unfortunately most malignant gliomas are resistant to chemotherapeutic agents and patients have a mean survival of 12 months after diagnosis [4]. The aggressive nature of glioma is attributed to intense cell proliferation, diffuse infiltration, and high resistance to apoptosis [5], [6], yet the factors that mediate glioma invasion and recurrence are still poorly understood. Therefore, there is an urgent need to identify critical carcinogenic pathways and discover new therapeutic targets for glioma [7].

ZEB2 (also known as SIP1) is a member of the Zfh1 family of 2-handed zinc finger/homeodomain proteins. The relevance of ZEB2 to tumor progression has been studied in several forms of human cancer and it has been associated with various clinicopathological features such as histological type, differentiation grade, and overall survival [8], [9], [10]. Recent reports highlighted that ZEB2 targeted by the miR-200 family [11], [12], [13] was closely related to Epithelial-to-Mesenchymal Transition (EMT), suggesting ZEB2 is a key factor in promoting the initiation and development of tumors.

In this study, we evaluated the expression of ZEB2 in human patient samples. Further, to explore its associated molecular mechanisms in glioma cells, we examined the effect of targeted silencing of ZEB2 gene on cell proliferation, EMT, and cell apoptosis using siRNA in vitro. These findings will be useful in identifying potential candidates for targeted therapeutic intervention of glioma.

## Results

### Immunohistochemical Analysis of ZEB2 Protein Expression in Glioma and Normal Brain Tissues

**Figure 1:**
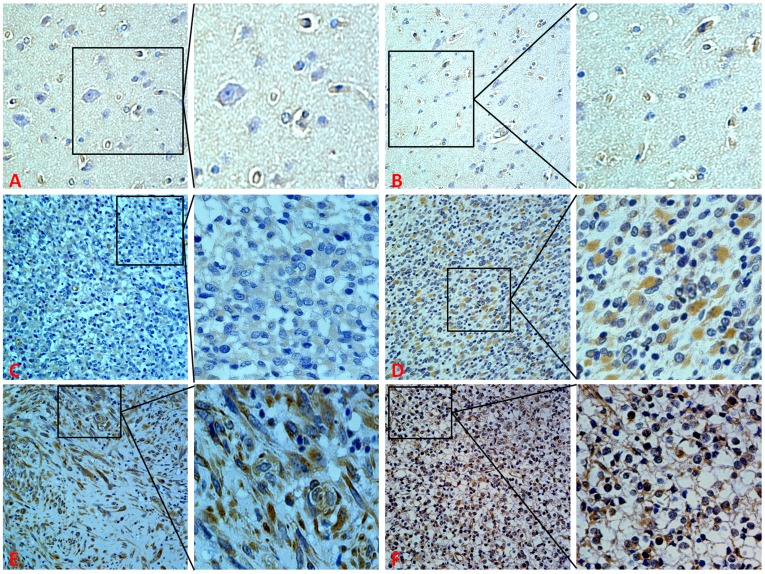
Expression of ZEB2 in glioma and normal brain tissues. A and B. Weak expression of ZEB2 in normal brain tissues. **C.** Weak expression of ZEB2 in glioma samples. **D.** Medium expression of ZEB2 in glioma samples. **E and F.** Strong staining of ZEB2 in glioma samples. (Original magnification 400×).

Expression levels and subcellular localization of ZEB2 protein was measured in 90 archived paraffin-embedded glioma samples and 10 normal brain tissues using immunohistochemical staining (Figure 1A–F). ZEB2 was highly expressed in 65.6% (59/90) of glioma samples compared to only 20.0% (2/10) of normal brain samples, which was a statistically significant difference (*P*<0.001) (Table 1).

**Table 1:** Protein expression of ZEB2 between glioma and normal brain tissues.

Group		Protein expression		*P* value
	Cases	High expression	Low expression	
cancer	90	59	31	
normal	10	2	8	0.000

The relationship between clinicopathologic characteristics and ZEB2 expression in individuals with glioma are summarized in Table 2. No significant association between ZEB2 expression levels and patient’s age or sex was observed in any of the 90 glioma cases. However, we observed that the expression of ZEB2 was positively correlated with the status of pathology classification (WHO I-II vs. WHO III-IV) (*P* = 0.024) in glioma patients (Table 2).

### Downregulation of ZEB2 Inhibits Glioma Cell Proliferation, Migration and Invasion in U251 and U87 Cells

**Figure 2:**
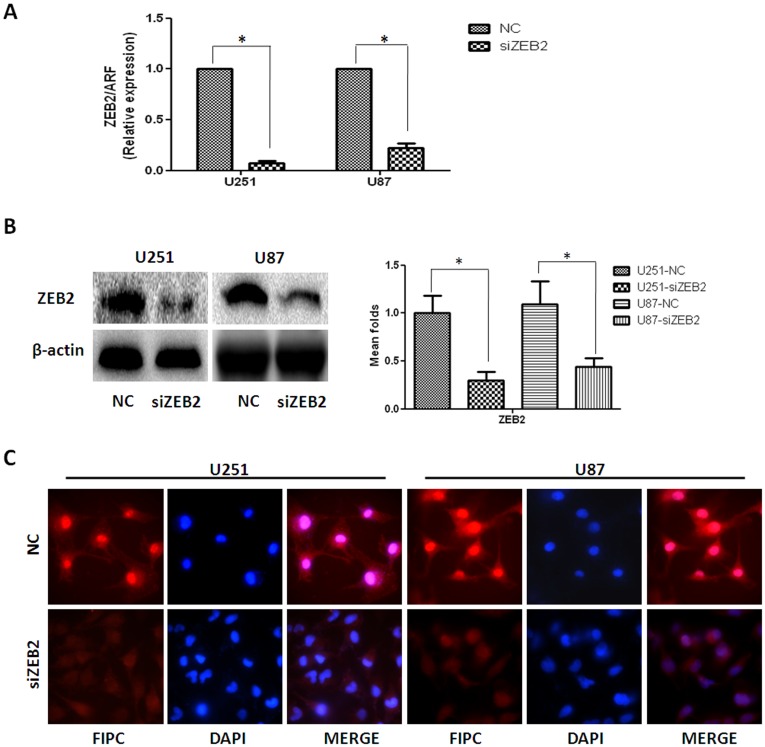
Effect of siRNA interference on ZEB2 expression in human glioma cell lines U251 and U87. Different treatments included negative control (NC) and siZEB2-transfected groups (siZEB2). **A.** RT-PCR shows transcriptional levels of the ZEB2 gene 48 hr post transfection and ARF was used as a loading control. The arbitrary units were plotted using mean ± SD of at least three individual repetitions. **B.** Western blot showing protein expression levels in NC and siZEB2 treatments. At 72 hr post transfection, cells were harvested and whole cell lysates prepared using RIPA buffer. The representative image of three different repetitions is shown. β-actin served as a loading control. Bar graph shows the relative expression of protein among the groups. Data are presented as mean ± SD for three independent experiments. **C.** Immunofluorescence study using blinded analysis showing the expression of ZEB2 in NC and siZEB2-treated U251 and U87 cells at 48 hr post-transfection (Original magnification 1000×). *P<0.05, statistically significant difference.

To explore the role of ZEB2 in glioma we utilized two human cell lines established from high-grade tumors (U251 and U87 cells). Transcriptional levels of ZEB2, as assessed by RT-PCR, were decreased up to 92.8% and 77.6% in siZEB2-transfected U251 cells and U87 cells compared to the negative control (si-NC treated) groups respectively (*P*<0.05) (Figure 2A). Consistent results for protein levels were observed by western blot (Figure 2B) and immunofluorescence at 48 hr (Figure 2C).

**Table 2:** Correlation between the clinicopathologic characteristics and expression of ZEB2 protein in glioma.

			ZEB2(%)		
Characteristics		n	High-expression	Low-expression	*P*
Gender					
	Male	53	36(67.9%)	17(32.1%)	
	Female	37	23(62.2%)	14(37.8%)	0.571
Age					
	≥50	48	32(66.7%)	16(33.3%)	
	<50	42	27(64.3%)	15(35.7%)	0.813
WHO Grade					
	I	10	4(40.0%)	6(60.0%)	
	II	25	14(56.0%)	11(44.0%)	
	III	26	18(69.3%)	8(30.7%)	
	IV	29	23(79.3%)	6(20.7%)	0.024

**Figure 3:**
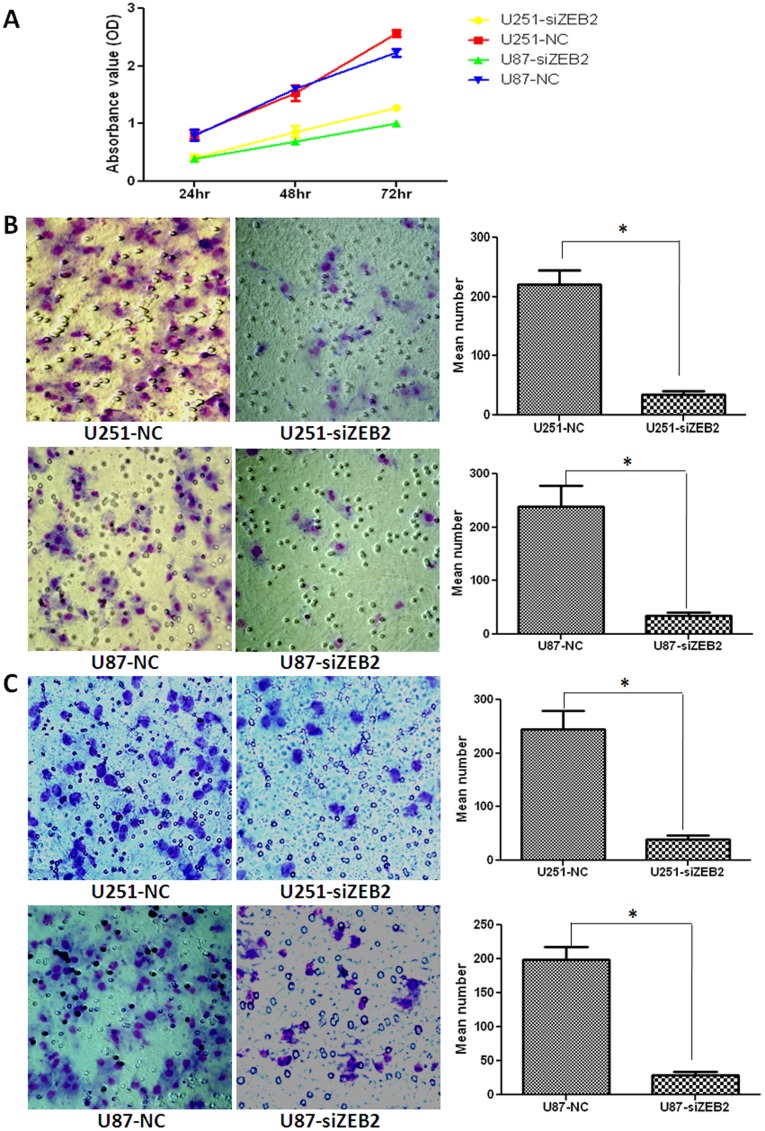
Downregulation of ZEB2 inhibits glioma cell proliferation, migration and invasion in vitro. A. Effect of ZEB2 knockdown on U251 and U87 cell proliferation as measured by MTT assay. Absorbance was read at 490 nm with averages from triplicate wells. **B and C.** Downregulation of ZEB2 dramatically reduced U251 and U87 cell migration and invasion in vitro. Data are presented as mean ± SD for three independent experiments (Original magnification 400×). *P<0.05, statistically significant difference.

The growth curves determined by MTT assay showed that downregulation of ZEB2 significantly inhibited cell proliferation of these two cell lines (Figure 3A). To examine the effect of ZEB2 on cell migration, siZEB2-transfected U251 and U87 cells were cultured on transwell apparatus. After 8 hr incubation, the percentage of migrated cells was significantly less in both siZEB2-treated groups (for both *P*<0.05) (Figure 3B). Using a boyden chamber coated with matrigel, we determined changes in cell invasiveness after 8 hr incubation. When compared with the negative control groups, siZEB2-transfected U251 and U87 cells both exhibited significantly decreased invasiveness (both *P*<0.05) (Figure 3C).

### ZEB2 Downregulation Promotes Expression of E-cadherin but Suppresses EMT Progression in Glioma Cells

**Figure 4:**
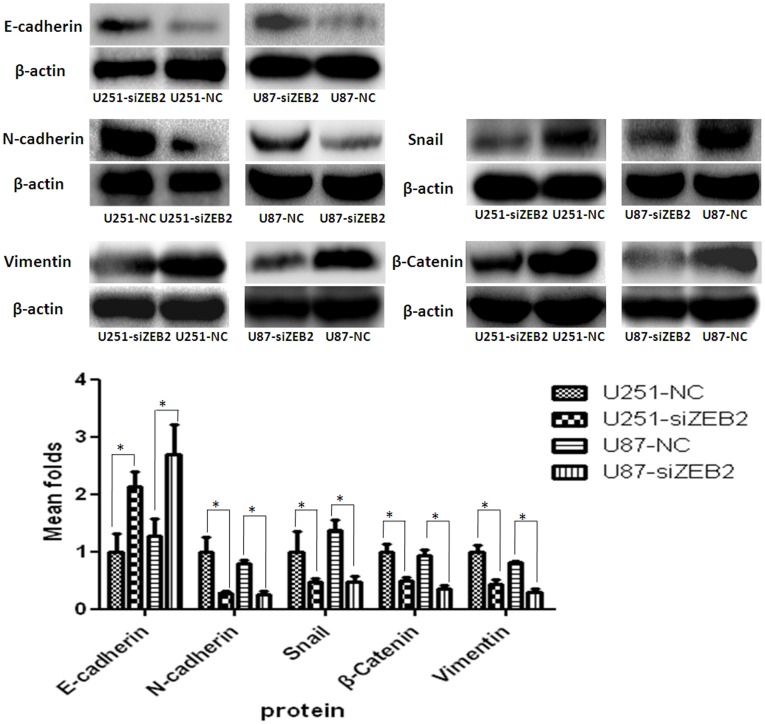
ZEB2 downregulation promotes expression of E-cadherin but suppresses EMT progression in glioma cells. ZEB2 reduction enhanced expression of E-cadherin and expression level changes of N-cadherin, Snail, β-Catenin and Vimentin in U251 and U87 cells at 72 hr post-transfection. β-actin was used as a loading control. Bar graph shows the relative expression of protein among the groups. Data are presented were presented as mean ± SD for three independent experiments. *P<0.05, statistically significant difference.

**Figure 5:**
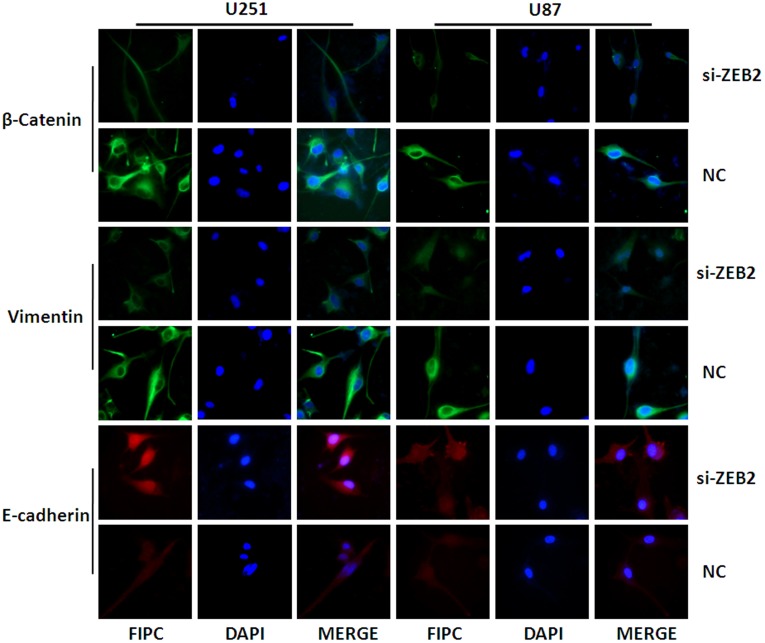
Immunofluorescence of EMT related regulators in NC and siZEB2-treated U251 and U87 cells at 48 hr post-transfection. High expression of β-Catenin and Vimentin and low E-cadherin expression in U251 and U87 cells was observed. After transfection with siZEB2 in both glioma cell lines, the expression of β-Catenin and Vimentin were decreased while E-cadherin was increased. (Original magnification 400×).

Examination of the protein levels indicated that E-cadherin was significantly upregulated after siZEB2 transfection in both lines (Figure 4). Similarly, the expression of EMT-related regulators, such as N-Cadherin, Vimentin, β-Catenin and Snail were decreased after ZEB2 knockdown (Figure 4). By immunofluorescence U251 and U87 cells exhibited high expression levels of β-Catenin and Vimentin. The fluorescence intensity indicated a significant decrease in the β-Catenin and Vimentin expression and increase in the E-cadherin expression after 48 hr siZEB2 treatment (Figure 5).

### Downregulation of ZEB2 Expression Induces Cell Cycle Arrest at G1/S Phase by Regulating Cell Cycle-related Genes *in vitro*

**Figure 6:**
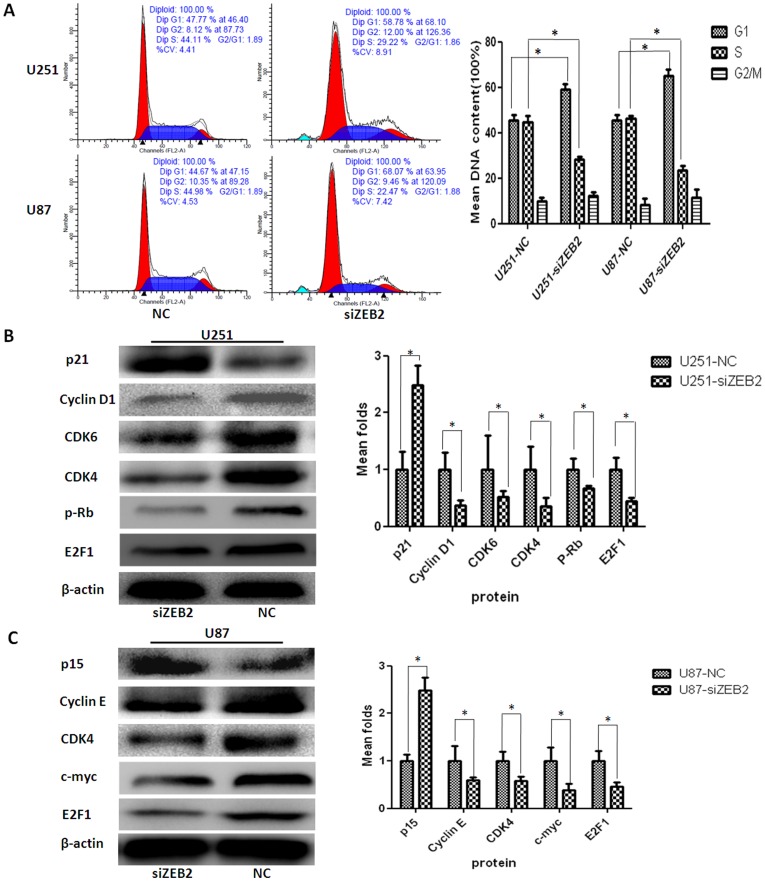
Downregulation of ZEB2 expression induced cell cycle arrest at G1/S phase by regulating cell cycle-related genes. **A.** The cell cycle distribution in siZEB2 treated and NC groups of U251 and U87 glioma cells by FACS Caliber cytometry. Histograms showing G1/S phase arrest and a decline in S phase in U251 and U87 cells after knocking down ZEB2 expression through targeted siRNA transfection. **B and C.** The protein levels of several key cell cycle regulators was analyzed by western blot. Reduced ZEB2 expression significantly inhibited cell cycle progression in U251 and U87 glioma cells. β-actin was used as a loading control. Bar graph shows the relative expression of protein among the groups. Data are presented were presented as mean ± SD for three independent experiments. *P<0.05, statistically significant difference.

To study the link between cell cycle control and ZEB2 in glioma cells, we first assessed the cell cycle distribution by flow cytometry at 48 hr post transfection between negative control or siZEB2 treated cells. As shown in Figure 6A, cells exhibited a significant increase in the cell fraction in G1 phase (U251-NC 45.50±2.41%, U251-siZEB2 59.20±2.35%, U87-NC 45.37±2.33%, U87-siZEB2 65.06±2.88%, *P*<0.05) and a corresponding reduction in the fraction of cells in S phase (U251-NC 44.83±2.52%, U251-siZEB2 28.40±0.93%, U87-NC 46.35±1.20%, U87-siZEB2 23.37.06±2.17%, *P*<0.05), while the percentage of cells in G2-M phase was unaffected by ZEB2 inhibition.

To further study the mechanism by which ZEB2 regulates cell cycle, we examined the protein levels of several key regulators of cell cycle progression. CDK4 and E2F1 protein levels were decreased in siZEB2-treated both for U251 and U87 cells. However, compared to negative control groups, p21 protein levels only increased in U251 cells but not in U87 cells, while the expression of P15 was increased in U87 cells but not in U251 cells at 72 hr post transfection (Figure 6B–C). Additionally, CDK6, p-Rb, and Cyclin D1 were decreased in siZEB2 treated U251 while c-myc and Cyclin E decreased in U87 cells. The expression levels of other significant cell cycle regulators including p27, p16 were unaffected after ZEB2 knockdown (Figure S1).

### ZEB2 Downregulation Induces Apoptosis by the Activation of Caspase-3 in Glioma Cells

**Figure 7:**
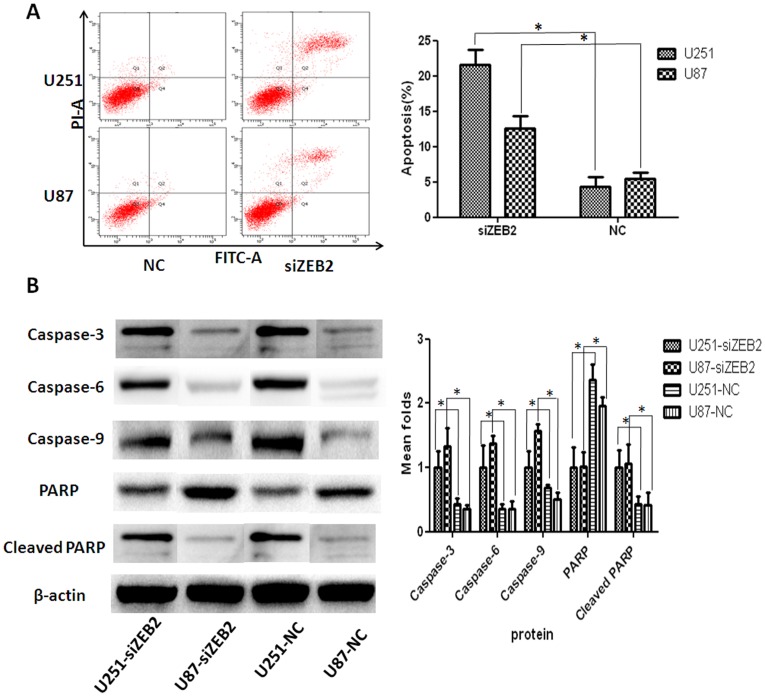
ZEB2 downregulation induces apoptosis by the activation of Caspase-3 in glioma cells. **A.** Apoptosis in U251 and U87 cells was measured by Annexin V-FITC/propidium iodide (PI) staining following siZEB2 or control treatment. Early apoptotic cell populations were significantly increased (P<0.01) after siZEB2 transfection. **B.** Western blot analysis for antiapoptotic PARP and effector Caspase-3, Caspase-6 and Caspase-9. Decreased PARP and increased caspase-3,-6,-9, and Cleaved PARP expression were observed in U251 and U87 cells after ZEB2 downregulation at 72 hr post transfection. β-actin was used as a loading control. Bar graph shows the relative expression of protein among the groups. Data are presented were presented as mean ± SD for three independent experiments. *P<0.05, statistically significant difference.

The rate of cellular apoptosis in U251 and U87 cells was examined using flow cytometric analysis with Annexin V staining. The cellular apoptosis rate of U251 cells was 21.63±2.10% in siZEB2-treated and 4.03±1.45% in negative control at 48 hr post transfection. In U87 cells, apoptotic rates were 5.49±0.79% in untransfected controls, while 12.53±1.86% in siZEB2-treated (Figure 7A). Thus, downregulation of ZEB2 significantly induced apoptosis in both the U251 and U87 cells. We next assessed the mechanism by which ZEB2 regulates apoptosis. Using western blot analysis, we found that Caspase-3, Caspase-6, Caspase-9 and Cleaved-PARP protein levels increased while PARP protein decreased in both siZEB2 treated U251 and U87 cells compared to negative controls (Figure 7B).

## Discussion

The expression of ZEB2 has been reported in different tumors [8], [9], [10]. Elevated expression of ZEB2 is associated with degree of malignancy, rapid cell proliferation, and poor patient survival in different tumors [8], [9], [14], suggesting that ZEB2 expression may have significant value as a biomarker in glioma patients.

In the present study, we firstly confirmed that ZEB2 mRNA levels were higher in glioma samples than that in normal brain tissues (Figure S2). Further, we found that ZEB2 was mainly localized in the nucleus and cytoplasm of glioma tissues while predominantly weakly expressed in cytoplasm in normal brain tissues by immunohistochemistry. Consistent with mRNA levels, ZEB2 protein expression was significantly elevated in glioma samples compared to normal brain tissues. Furthermore, we also observed that the expression level of ZEB2 was positively correlated with tumor grading as there was a significant difference between high and low grade gliomas. These data strongly imply an oncogenic role of ZEB2 in glioma tumorigenesis.

The biological functions of ZEB2 found in this study provided a mechanistic basis for the pathological and clinical observations. In agreement with a previous study [15], we found that knockdown of ZEB2 expression inhibited cell proliferation, migration, and invasion of glioma cells. However, there is little knowledge of the relationship between ZEB2 and cell cycle progression or apoptosis in glioma cells. In our study, we demonstrated that high ZEB2 expression not only regulated EMT, but also mediated the cell cycle progression and apoptosis in glioma cell lines.

ZEB2 overexpression is characterized by a full-scale shift in phenotype indicative of EMT [16], and is involved in cell migration and invasion which are key steps in the progression of glioma [17]. The loss of E-cadherin and overexpression of mesenchymal cell markers such as N-cadherin, Vimentin are hallmarks of EMT [18]. Consistent with Xia et al’s report [15], our data indicated that knockdown of ZEB2 resulted in restoration of E-cadherin expression and suppression of Vimentin expression in glioma cells. Furthermore, we observed that ZEB2 downregulation also inhibited the expression of other mesenchymal cell markers such as N-Cadherin, β-Catenin and transcription factor Snail.

ZEB2 regulation of cell growth has been reported through cell colony formation assays in glioma [15]. In this study, we also observed that lost expression of ZEB2 reduced cell proliferation using an MTT assay. Decreased expression of ZEB2 reduced the cell population at S phase, suggesting an inhibition of cell cycle progression from G1 to S phase, while the percentage of cells in G2-M phase was unaffected by ZEB2 inhibition. This might be partly attributed to the suppressive effect of ZEB2 on cell cycle progression. This process is partly dependent on the tightly regulated activity of Cyclin dependent kinases (CDKs). Cyclin D/CDK4/6 activity occurs in mid-late G1 phase, upstream of CDK2/Cyclin E activity. Both of these pathways are required for hyperphosphorylation of the retinoblastoma gene product (pRb) [14]. One of the cell cycle regulatory pathways most often affected in cancer is the G1 checkpoint, which is controlled by a complex network of Cyclin D, CDKs, Rb and Rb-related proteins. E2F1 is a key downstream target of pRb [19]. There is a significant correlation between high levels of E2F1 protein and high-grade glioma [20]. E2F1 as well as c-myc play a key roles in G1/S transition [21], [22]. Increased expression of c-myc is associated with neoplastic transformation and angiogenesis in glioma [23], [24]. In this investigation, we found that the downregulation of ZEB2 by siRNA induced increases of p21 in U251 cells and p15 in U87 cells. Meanwhile, siZEB2 decreased CDK4/6, Cyclin D1, E2F1 protein levels U251 cell, and CDK4, Cyclin E, E2F1 and c-myc in U87 cells. These results are partially consistent with Yang et al’s report [20]. The changes of CDK4/6 and p21 mediated by ZEB2 knockdown in the U251 glioma cell line suggest potential avenues to target these proteins in tumors. Similarly, in U87 cells, c-myc, Cyclin E, and p15 protein expression were mediated by ZEB2. Because of the difference sources of U251 [25] and U87 [26] cell lines, we considered that siZEB2 treatment induced cell cycle arrest at G1/S phase through different pathways, and this may be dependent on tumor types. Interestingly, the expression levels of other significant cell cycle regulators including p27 and p16 did not change after ZEB2 knockdown which suggests similar regulatory effects on the cell cycle machinery mediated by ZEB2 in glioma cells.

Apoptosis is a well-orchestrated cellular mechanism that balances cell proliferation and cell death. Recent evidence indicates that ZEB transcription factors may also regulate apoptosis and senescence separately from regulation of EMT [27], [28], [29]. DNA breakage and activation of DNA damage response often leads to apoptosis or senescence. PARP is activated during DNA strand break repair [30], [31], and has been previously demonstrated to be requiremed for DNA damage-induced NF-κB [32]. Inhibition of PARP degradation leads to persistent NF-κB DNA binding, increased anti-apoptotic gene expression and a protection against cell death [33]. Our data indicate that reduced expression of ZEB2 significantly induced apoptotic cell death in both the U251 and U87 cells. In corroboration with these results, siZEB2 treatment resulted in the elevation of Cleaved-PARP, Caspase-3, Caspase-6, and Caspase-9 as well as reduced PARP levels in U251 and U87 cells.

We hypothesize that ZEB2 regulates cell cycle progression, migration, invasion, and apoptosis in glioma through at least two molecular mechanisms. ZEB2 may contribute to tumor progression by protecting cancer cells from apoptosis and DNA damage by activating NF-κB, which can prevent cell death in glioma [34] and result in downregulation of E-cadherin mediated classical EMT [35]. Meanwhile, repressed expression of Caspase-3, -6, -9 and enforced expression of PARP from ZEB2 can enhance DNA repair and cell cycle progression. ZEB2 may also function as a transcriptional promoter as shown for the Smad-dependent TGF-β signaling pathway [36], [37], ZEB2 may also reduce expression of the cell cycle inhibitors p15 and p21, and promote G1 to S phase cell cycle progression by mediating the activity of CDK4/6, Cyclin D1, and Cyclin E, which will eventually result in hyperphosphorylation of RB, releases of E2F1 and overexpression of the mitogenic transcription factor c-myc.

In summary, ZEB2 expression may have significant value as an unfavorable progression indicator for glioma patients. We provide compelling evidence that decreased expression of ZEB2 inhibits cell proliferation, migration, invasion, and induces cell death through multiple pathways involving in cell cycle, apoptosis, and EMT.

## Materials and Methods

### Sample Collection

Ten fresh normal brain tissues sample and ten glioma samples were collected from the Nanfang Hospital of Southern Medical University, Guangzhou, China, at the time of diagnosis before any therapy. All fresh samples were immediately preserved in liquid nitrogen. Ninety archived paraffin-embedded glioma and 10 normal brain tissues samples were obtained from the Nanfang Hospital of Southern Medical University, Guangzhou, China. These cases were from 53 males and 37 females with age ranging from 13 to 68 years (median 42.1 years). All specimens had confirmed pathological diagnosis and were classified according to the World Health Organization (WHO) criteria.

### Ethics Statement

For the use of these clinical materials for research purposes, there were 3 children participants involved in our study, and we obtained written informed consent from their guardians or next of kin. We obtained all written informed consent from adult patients directly. Prior consents from all patients and approval from the Ethics Committees of Nanfang Hospital were obtained.

### Immunohistochemistry Staining

The details of immunohistochemistry methods were used as described previously [38]. The anti-human ZEB2 antibody (1∶150) (Bioworld, Inc,USA) was used. The immunohistochemically stained tissue sections were reviewed and scored separately by two pathologists blinded to the clinical parameters. Expression of ZEB2 in the nucleus and in the cytoplasm was independently evaluated. For cytoplasmic staining, the score was evaluated according to the sum of cytoplasm staining intensity and the percentage of positive staining areas of cells. The staining intensity was scored as previously described [38](Materials & Methods S1).

### Cell Culture

The human glioma cell lines U251 and U87 were purchased from the Chinese Academy of Sciences (Shanghai, China) and grown in Dulbecco’s modified Eagle’s medium (DMEM) (Hyclone, Logan, UT) supplemented with 10% fetal calf serum (ExCell, Shanghai, China). All cell lines were cultured at 37°C in a humidified atmosphere of 5% CO_2_.

### RNA Isolation and Detection of ZEB2 mRNA Levels by Real-time PCR

Total RNA from fresh ten normal brain tissues sample, ten glioma samples and glioma cells was isolated by using Trizol reagent (Invitrogen, Carlsbad, CA) according to the manufacturer’s instructions. The ImProm-II Reverse Transcription kit (Promega Corporation, Madison, WI) along with 2 mg of total RNA and poly-dT primers were used for synthesis of cDNA. To determine the RNA transcript levels from cDNA, Real-time PCR was carried out using a MX3000P instrument (Stratagene, La Jolla, CA, USA) and SYBR® Premix Ex Taq™ kit (Takara, Otsu, Japan) as described previously [39] to detect the mRNA level of ZEB2. The sequence for sense primer was 5′-GGCGCAAACAAGCCAATCCCA-3′, and for antisense primer was 5′-TTCACTGGACCATCTACAGAGGCTT-3′. ARF gene was used as an internal control using the sense primer 5′-ATCTGTTTCACAGTCTGGGACG-3′ and antisense primer 5′-CCTGCTTGTTGGCAAATACC-3′. PCR cycling conditions were 95°C for 10 min to activate DNA polymerase, followed by 45 cycles of 95°C for 15 s, 55°C for 15 s, and 72°C for 10 s. Specificity of amplification products was confirmed by melting curve analysis. Independent experiments were done in triplicate.

### Transient Transfection with siRNAs

Small-interfering RNA (siRNA) were designed and synthesized by Guangzhou RiboBio (RiboBio Inc, China). Three siRNAs targeting on ZEB2 gene were designed and synthesized, the most effective siRNA (siZEB2) identified by Real Time-PCR was applied for the further experiments. The sequence of siZEB2 is: sense: 5′- GGACACAGGUUCUGAAACA dTdT-3′; anti-sense: 3′- dTdT CCUGUGUCCAAGACUUUGU-5′. The sequence of si-negative control (si-NC) was also designed by RiboBio (RiboBio Inc, China). Twenty-four hours prior to transfection, U251 or U87 cells were plated onto a 6-well plate or a 96-well plate (Nest, Biotech,China) at 40–60% confluence. Cells were then transfected by incubation with siZEB2 (si-NC as a control) at final concentrations of 50 nM with TurboFectTM siRNA Transfection Reagent (Fermentas). The medium was not replaced after transfection according to the manufacturer’s protocol. Cells were collected after 48 hr for the following assays or after 72 hr for RNA and protein extraction (Figure S3).

### Cell viability and Proliferation Assay

Cell proliferation was analyzed using 3-(4,5-dimethylthiazol-2-yl)-2, 5-diphenyltetrazolium bromide (MTT) assay. Cells were seeded in 96-well plates at a density of 700 cells/well. The cells were incubated for 1, 2 or 3 days. Twenty microliters of MTT (5 mg/ml) (Sigma, St. Louis, MO) was added to each well and incubated for 4 hr. At the end of incubation, the supernatants were removed, and 150 µl of DMSO (dimethyl sulfoxide) (Sigma, St. Louis, MO) was added to each well. The absorbance value (OD) of each well was measured at 490 nm. For each experimental condition, eight wells were used. Experiments were performed thrice.

### Cell migration and Invasion Assays

For cell migration assays, 1×10^4^ cells in 100 µl DMEM medium without FCS were seeded on a fibronectincoated polycarbonate membrane insert in a transwell apparatus (Costar, MA). In the lower chamber, 600 µl DMEM with 10% FCS was added as a chemoattractant. After the cells were incubated for 8 hr at 37°C in a 5% CO_2_ atmosphere, the insert was washed with PBS, and cells on the top surface of the insert were removed with a cotton swab. Cells adhering to the lower surface were fixed with methanol, stained with Giemsa solution and counted under a microscope in five predetermined fields (200×). All assays were independently repeated at least thrice.

For cell invasion assays, the procedure was similar to the cell migration assay, except transwell membranes were precoated with 24 µg/µl Matrigel (R&D Systems, USA) and the cells were incubated for 8 hr at 37°C in a 5% CO2 atmosphere. Cells adhering to the lower surface were counted the same way as the cell migration assay.

### Cell Cycle Analysis

For cell cycle analysis, cells were seeded on 10-cm–diameter plates in DMEM containing 10% FCS. After incubation for 48 hr, a total of 5×10^6^ cells were harvested, rinsed with cold PBS and fixed with 70% ice-cold ethanol for 48 hr at 4°C. Fixed cells were rinsed with cold PBS followed by incubation with PBS containing 10 µg/ml propidium iodide and 0.5 mg/ml RNase A for 15 min at 37°C. The DNA content of labeled cells was acquired using FACS caliber flow cytometry (BD Biosciences). Each experiment was performed in triplicate.

### Apoptosis Assay

Apoptosis was measured by using an Annexin V/fluorescein isothiocyanate (FITC) apoptosis detection kit (Keygen, China). Briefly, cells cultured in 6-cm dishes were trypsinized, washed, stained with FITC-conjugated anti-Annexin V antibody under darkness for 15 min at room temperature, and then analyzed by flow cytometry (BD Biosciences). Each experiment was performed in triplicate.

### Immunofluorescence

U251 and U87 cells were seeded on coverslips in 6-well plate and cultured overnight. Subsequently cells were fixed in 3.5% paraformaldehyde, permeabilized in KB solution and 0.2% Triton X-100 at room temperature. After the blocking solution was removed, cells were incubated with a primary antibody (diluted in KB) 30–45 min at 37°C and subsequently washed with KB twice. After incubating 30–45 min at 37°C with secondary antibody (diluted in KB), cells were washed with KB again, then mounted onto slide with mounting solution containing 0.2 µg/ml DAPI and seal with nail polisher. Slides were stored at 4°C in a dark box and observed under a fluorescent microscope.

### Western Blot Analysis

Cells were lysed in RIPA Buffer (50 mM Tris-HCl pH 8.0, 1 mM EDTA pH 8.0, 5 mM DTT, 2% SDS), and protein concentration was determined by using BCA assay (Beyotime Inc, China). Total protein (30 µg) was resolved using a 10% SDS-PAGE gel and electro-transferred to polyvinylidene fluoride membranes (Invitrogen, Carlsbad, CA), and blocked with 5% nonfat dry milk in Tris-buffered saline, pH 7.5. Membranes were immunoblotted overnight at 4°C (Materials & Methods S2).

### Statistical Analysis

All quantified data represented an average of at least triplicate samples or as indicated. SPSS 13.0 and Graph Pad Prism 4.0 software were used for statistical analysis. Data are represented as mean ± S.E.M. One-way ANOVA or two-tailed Student’s t-test was used for comparisons between groups. Chi-square test or Fischer’s were used to identify differences between categorical variables. Differences were considered statistically significant when *P*<0.05.

## Supporting Information

Figure S1 The protein expression levels of some key cell cycle-related genes after knockdown of ZEB2 in glioma cells. The protein levels of several key regulators of cell cycle was analyzed by western blotting. In U251 cells, the expression levels of p27, p16, p15, Cyclin E, c-myc were not significantly differences between the siZEB2 treated groups and the NC groups. Similarly, the expression levels of some cell cycle-related genes, such as CDK6, p27, p21, p16, Cyclin D1, and p-Rb, were not significantly differences between the siZEB2 and NC groups in U87 glioma cells. β-actin is used as a loading control. *P<0.05, statistically significant difference.
(TIF)

Figure S2 The mRNA levels of ZEB2 in normal brain tissues and glioma samples. The mRNA levels of ZEB2 from ten normal brain tissues sample and ten glioma samples were analyzed by RT-PCR. Data showed that the mRNA levels of ZEB2 in glioma were higher than that of normal brain tissues (0.8579±0.05737/0.4131±0.05357). *P<0.05, statistically significant difference.
(TIF)

Figure S3 Effect of siRNA interference on ZEB2 expression in human glioma cell lines U251 and U87 analyzed by Real-time PCR. RT-PCR shows transcriptional levels of the ZEB2 gene after transfection using three different RNAi targeted ZEB2, and ARF was as a loading control. The sequence of siRNA-1 is: sense: 5′- GGACACAGGUUCUGAAACA dTdT-3′; anti-sense: 3′- dTdT CCUGUGUCCAAGACUUUGU-5′; The sequence of siRNA-2 is: sense: 5′- CUGCAAGGCUGAAGAAAUU dTdT-3′; anti-sense: 3′- dTdT GACGUUCCGACUUCUUUAA-5′; The sequence of siRNA-3 is: sense: 5′- CAAAUAAUCUGGACAACAA dTdT-3′; anti-sense: 3′- dTdT GUUUAUUAGACCUGUUGUU-5′. **A.** 24 h post-transfection in U251 cells. **B.** 48 h post-transfection in U251 cells. **C.** 24 h post-transfection in U87 cells. **D.** 48 h post-transfection in U87 cells. The arbitrary units were plotted using mean ± SE of at least three individual repetitions. *P<0.05, statistically significant difference.
(TIF)

Materials & Methods S1 Evaluation of staining
(DOC)

Materials & Methods S2 Western blot
(DOC)
