# Expression of Concern: ZEB2 Mediates Multiple Pathways Regulating Cell Proliferation, Migration, Invasion, and Apoptosis in Glioma

**DOI:** 10.1371/journal.pone.0231386

**Published:** 2020-04-01

**Authors:** PLOS ONE

After publication of this article [1], concerns were raised about the identity of one of the cell lines used in the study, the reporting of sequence information, and the representation of the western blot data.

The authors provide the following clarifications and corrections:

The U87 glioblastoma cell line used in Figs 2–7 of this study was purchased from the Chinese Academy of Sciences (Shanghai, China). In 2018 cell line authentication was carried out by a third party company by STR profiling, which confirmed a match with the STR data of the U87 cell line in the databases of ATCC and DSMZ (S1 File). This U87 glioblastoma cell line has been reported elsewhere to be misidentified (https://web.expasy.org/cellosaurus/) [2], though likely to be derived from human glioblastoma [3].

**Fig 7:**
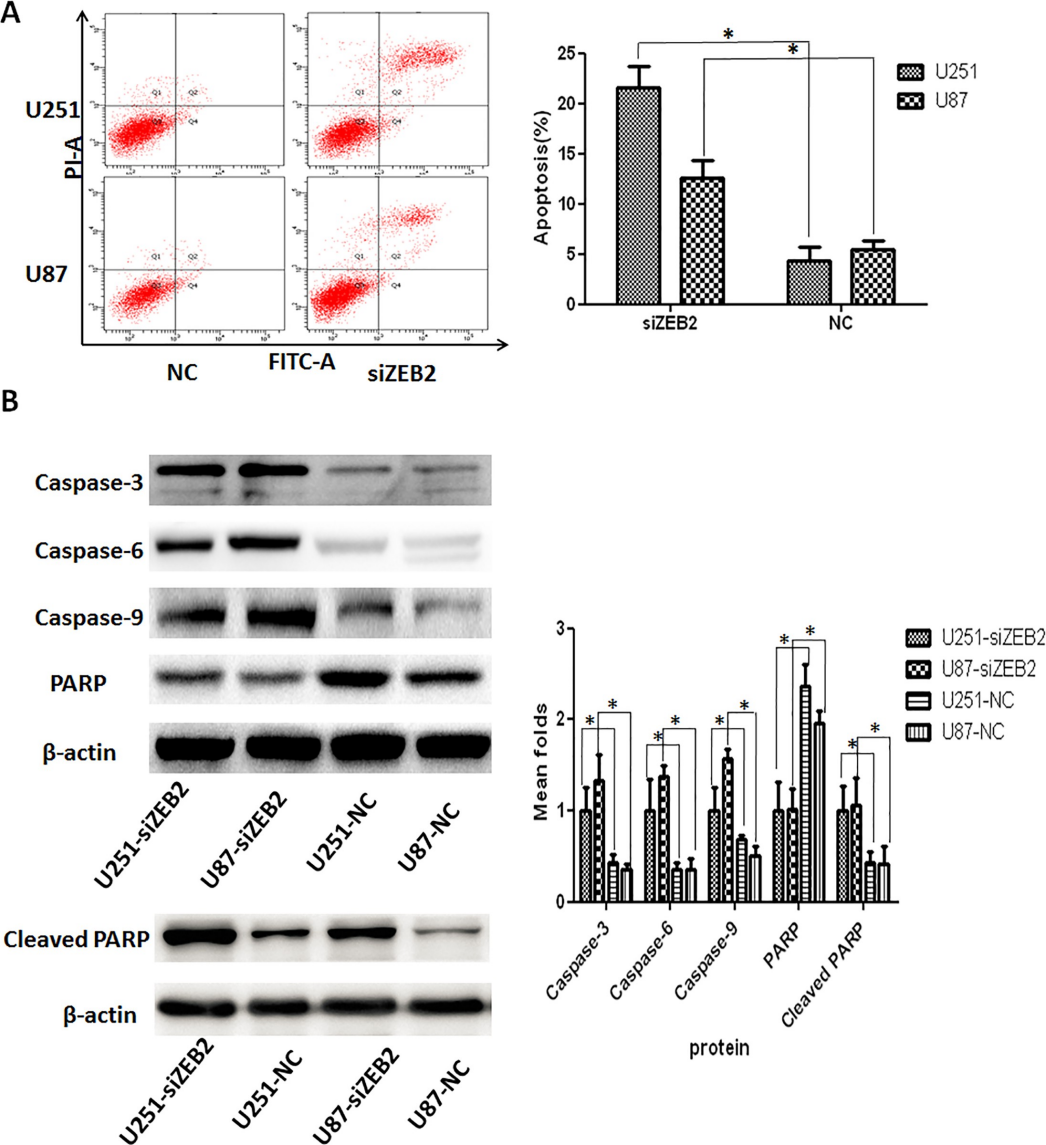
ZEB2 downregulation induces apoptosis by the activation of Caspase-3 in glioma cells. A. Apoptosis in U251 and U87 cells was measured by Annexin V-FITC/propidium iodide (PI) staining following siZEB2 or control treatment. Early apoptotic cell populations were significantly increased (P<0.01) after siZEB2 transfection. B. Western blot analysis for antiapoptotic PARP and effector Caspase-3, Caspase-6 and Caspase-9. Decreased PARP and increased caspase-3,-6,-9, and Cleaved PARP expression were observed in U251 and U87 cells after ZEB2 downregulation at 72 hr post transfection. β-actin was used as a loading control. Representative β-actin loading controls are shown for PARP and cleaved PARP; corresponding β-actin loading control panels are not available for Caspase-3, Caspase-6, and Caspase-9. Bar graph shows the relative expression of protein among the groups. Data are presented were presented as mean ± SD for three independent experiments. *P<0.05, statistically significant difference.

The control siRNA was designed and synthesized by RiboBio (RiboBio Inc, China) and the sequence information is commercially protected. The antisense strand of the siRNA targeting ZEB2 is 5’-UGUUUCAGAACCUGUGUCC-dTdT-3’. This was originally reported in the Methods section in 3’-5’ direction.

There is an error in Table 1 which reports a *P* value of 0.000. The correct *P* value is *P* = 0.005.

In Fig 4 the U87 sample western blots for Vimentin and β-Catenin were run on the same gel and share the same β-actin loading control. The U87 sample western blots for N-cadherin and Snail were run on the same gel; the N-cadherin and Snail bands shown for the U87-siZEB sample are taken from the same lane, and therefore share the same β-actin loading control. The original uncropped blots underlying Fig 4 are not available.

In Fig 7, splicing together of non-adjacent lanes was introduced during manuscript revision in response to review comments. Bands from the same gel were spliced together in order to rearrange the sample order for presentation purposes for Caspase-3, Caspase-6, Caspase-9, PARP, and Cleaved PARP, but the splicing was not clearly marked on the figure. Additionally, the β-actin and the associated labels were not rearranged in accordance with the amended lane order. The β-actin panel shown in the original figure was the loading control for the PARP blot only; all other proteins were blotted on different gels. The internal consistency of the samples was confirmed at the time of the experiments, but the corresponding loading control blots for the other proteins are no longer available, with the exception of cleaved PARP. The western blot for cleaved PARP was carried out in a later experiment, and due to an error during figure preparation, the incorrect blot was used for the cleaved PARP panel in the figure.

A revised Fig 7 is provided in which the original lane order is restored for Caspase-3, Caspase-6, Caspase-9 and PARP, which corresponds to the order of the β-actin panel and the sample labels; additionally, in the revised Fig 7, the cleaved PARP panel is replaced with the correct image, and its associated β-actin panel is provided. Corresponding β-actin loading control panels are not available for Caspase-3, Caspase-6, and Caspase-9.

The available underlying western blots for Fig 7 are provided here as S2 File.

The results of a repeat experiment carried out using a lentiviral shRNA vector to specifically and stably knock down the expression of ZEB2 in U87 cell lines are provided here as S3 File.

The *PLOS ONE* Editors issue this Expression of Concern to alert readers to the errors in the presentation of the western blots in Fig 7 and to the unavailability of both the individual-level data underlying the accompanying bar chart and the correct corresponding loading control images for the Caspase-3, Caspase-6, and Caspase-9 panels.

## Supporting information

S1 File U87 cell line authentication results. STR profiles of cell sample analyzed in December 2018; search results in ATCC and DSMZ databases showing a match to U87MG cell lines in both banks; and electrophoresis of gene COX1.
(PDF)

S2 File Western blots underlying Fig 7. (ZIP)

S3 File Repeat experiment. (ZIP)

## References

[1] Qi S, Song Y, Peng Y, Wang H, Long H, Yu X, et al (2012) ZEB2 Mediates Multiple Pathways Regulating Cell Proliferation, Migration, Invasion, and Apoptosis in Glioma. PLoS ONE 7(6): e38842 10.1371/journal.pone.0038842 22761708 PMC3383704

[2] Bairoch A. The Cellosaurus, a cell line knowledge resource. J. Biomol. Tech. 2018; 29: 25–38. 10.7171/jbt.18-2902-002 29805321 PMC5945021

[3] Allen M, Bjerke M, Edlund H, Nelander S, Westermark B. Origin of the U87MG glioma cell line: Good news and bad news. Science Translational Medicine. 2016 8 31 10.1126/scitranslmed.aaf6853 27582061

