# Supplementary figures and images for: ZEB2 Mediates Multiple Pathways Regulating Cell Proliferation, Migration, Invasion, and Apoptosis in Glioma
Source: PLoS One. 2012 Jun 26;7(6):e38842. doi: 10.1371/journal.pone.0038842 (PMC3383704; doi:10.1371/journal.pone.0038842)

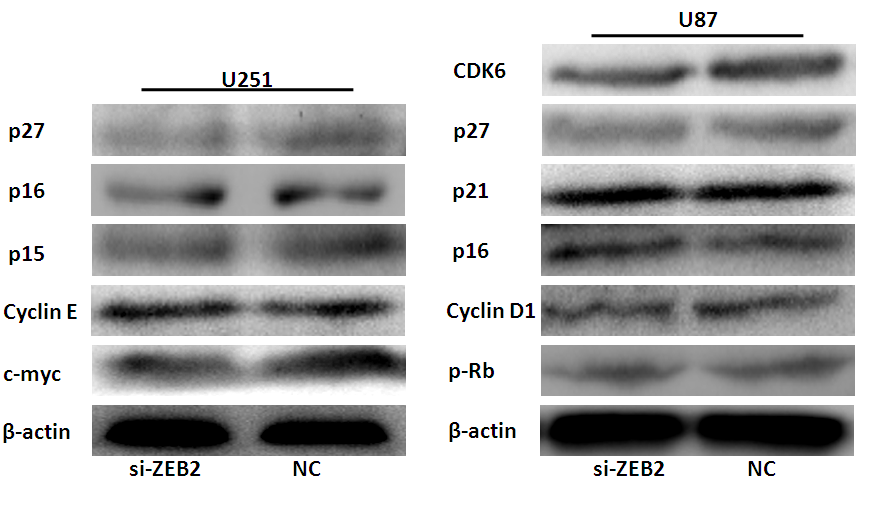

Supplement: Figure S1 — The protein expression levels of some key cell cycle-related genes after knockdown of ZEB2 in glioma cells. The protein levels of several key regulators of cell cycle was analyzed by western blotting. In U251 cells, the expression levels of p27, p16, p15, Cyclin E, c-myc were not significantly differences between the siZEB2 treated groups and the NC groups. Similarly, the expression levels of some cell cycle-related genes, such as CDK6, p27, p21, p16, Cyclin D1, and p-Rb, were not significantly differences between the siZEB2 and NC groups in U87 glioma cells. β-actin is used as a loading control. *P<0.05, statistically significant difference. (TIF) [file pone.0038842.s001.tif]

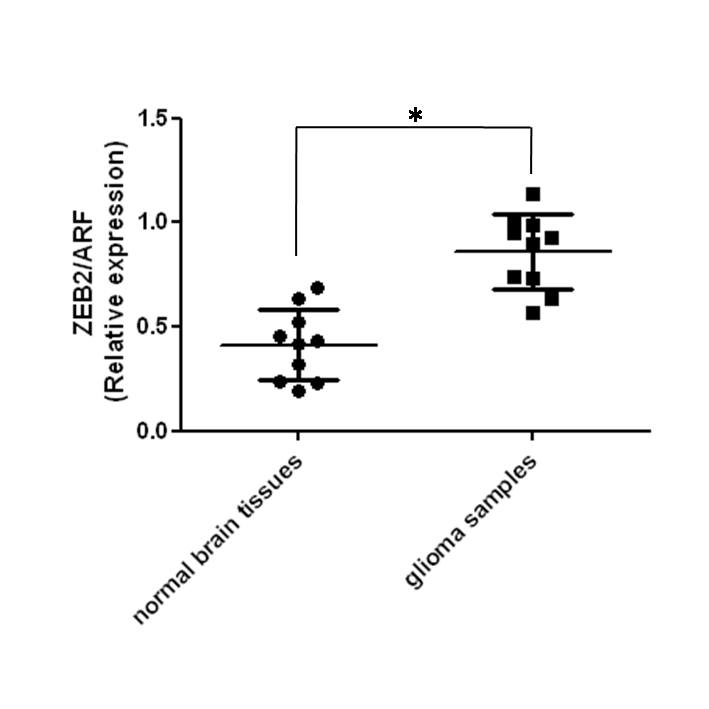

Supplement: Figure S2 — The mRNA levels of ZEB2 in normal brain tissues and glioma samples. The mRNA levels of ZEB2 from ten normal brain tissues sample and ten glioma samples were analyzed by RT-PCR. Data showed that the mRNA levels of ZEB2 in glioma were higher than that of normal brain tissues (0.8579±0.05737/0.4131±0.05357). *P<0.05, statistically significant difference. (TIF) [file pone.0038842.s002.tif]

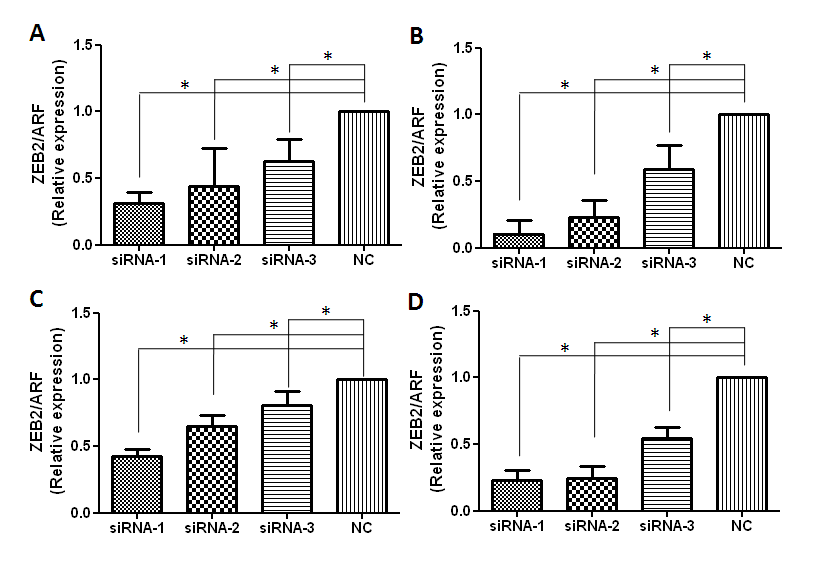

Supplement: Figure S3 — Effect of siRNA interference on ZEB2 expression in human glioma cell lines U251 and U87 analyzed by Real-time PCR. RT-PCR shows transcriptional levels of the ZEB2 gene after transfection using three different RNAi targeted ZEB2, and ARF was as a loading control. The sequence of siRNA-1 is: sense: 5′- GGACACAGGUUCUGAAACA dTdT-3′; anti-sense: 3′- dTdT CCUGUGUCCAAGACUUUGU-5′; The sequence of siRNA-2 is: sense: 5′- CUGCAAGGCUGAAGAAAUU dTdT-3′; anti-sense: 3′- dTdT GACGUUCCGACUUCUUUAA-5′; The sequence of siRNA-3 is: sense: 5′- CAAAUAAUCUGGACAACAA dTdT-3′; anti-sense: 3′- dTdT GUUUAUUAGACCUGUUGUU-5′. A. 24 h post-transfection in U251 cells. B. 48 h post-transfection in U251 cells. C. 24 h post-transfection in U87 cells. D. 48 h post-transfection in U87 cells. The arbitrary units were plotted using mean ± SE of at least three individual repetitions. *P<0.05, statistically significant difference. (TIF) [file pone.0038842.s003.tif]
