# Supplementary material for: ZEB2 Mediates Multiple Pathways Regulating Cell Proliferation, Migration, Invasion, and Apoptosis in Glioma
Source: PLoS One. 2012 Jun 26;7(6):e38842. doi: 10.1371/journal.pone.0038842 (PMC3383704; doi:10.1371/journal.pone.0038842)
Supplement: Materials & Methods S1 — Evaluation of staining (DOC) [file pone.0038842.s004.doc]

**Supplemental Materials and Methods**

S1 Evaluation of staining

The staining intensity was scored as 0 (negative), 1 (weak), 2 (medium) and 3 (strong) and the percentage of positive staining areas of cells was defined as a scale of 0 to 3 where 0 represents <10%, 1 is 10-25%, 2 is 26-75%, and 3 is ≥76%. For nuclear staining, the staining score was defined based on the sum of nuclear staining intensity and the number of positive nuclear staining. Nuclear staining intensity score was consistent with cytoplasm and positive nuclear staining scores were defined as follows: 0 represents <10%, 1 is 10-50%, 2 is 51-80%, and 3 is ≥80%. The sum of the cytoplasm and nuclear staining scores was used as the final staining score for ZEB2 (0-12). For statistical analysis, a final staining score of 0-6 or 7-12 was respectively considered to be low or high expression.
