# Supplementary material for: ZEB2 Mediates Multiple Pathways Regulating Cell Proliferation, Migration, Invasion, and Apoptosis in Glioma
Source: PLoS One. 2012 Jun 26;7(6):e38842. doi: 10.1371/journal.pone.0038842 (PMC3383704; doi:10.1371/journal.pone.0038842)
Supplement: Materials & Methods S2 — Western blot (DOC) [file pone.0038842.s005.doc]

**Supplemental Materials and Methods**

S2 Western blot

rabbit polyclonal anti-ZEB2 antibody(1:1000) (Bioworld Inc, USA), anti-Vimentin antibody(1:1500), anti-β-Catenin(1:1500), anti-Snail(1:1000), anti-E-Cadherin(1:1000), anti-N-Cadherin(1:1000), anti-Claudin-1(1:1000), anti- p27(1:500), anti- p15(1:500), anti-Phospho-Rb(1:500), anti-E2F1(1:500), anti-Cleaved Caspase-3(1:1000), anti- Cleaved Caspase-6(1:1000), anti-Cleaved Caspase-9(1:1000), anti-Cleaved-PARP (1:1000) and mouse anti-β-actin (1:500)(Santa Cruz, CA), polyclonal anti-CDK6(1:500), anti-CDK4(1:500), anti-C-myc(1:500), anti-Cyclin D1(1:500), anti-p21(1:500), anti-Cyclin E(1:500)(Cell Signaling Inc,USA). An HRP-conjugated anti-rabbit or anti-mouse IgG antibody was used as the secondary antibody (Zhongshan Inc, China)
