# Supplementary material for: Expression of Concern: ZEB2 Mediates Multiple Pathways Regulating Cell Proliferation, Migration, Invasion, and Apoptosis in Glioma
Source: PLoS One. 2020 Apr 1;15(4):e0231386. doi: 10.1371/journal.pone.0231386 (PMC7112199; doi:10.1371/journal.pone.0231386)
Supplement: S1 File — STR profiles of cell sample analyzed in December 2018; search results in ATCC and DSMZ databases showing a match to U87MG cell lines in both banks; and electrophoresis of gene COX1. (PDF) [file pone.0231386.s001.pdf]

Figure 1. STR profiles of U87 cell line

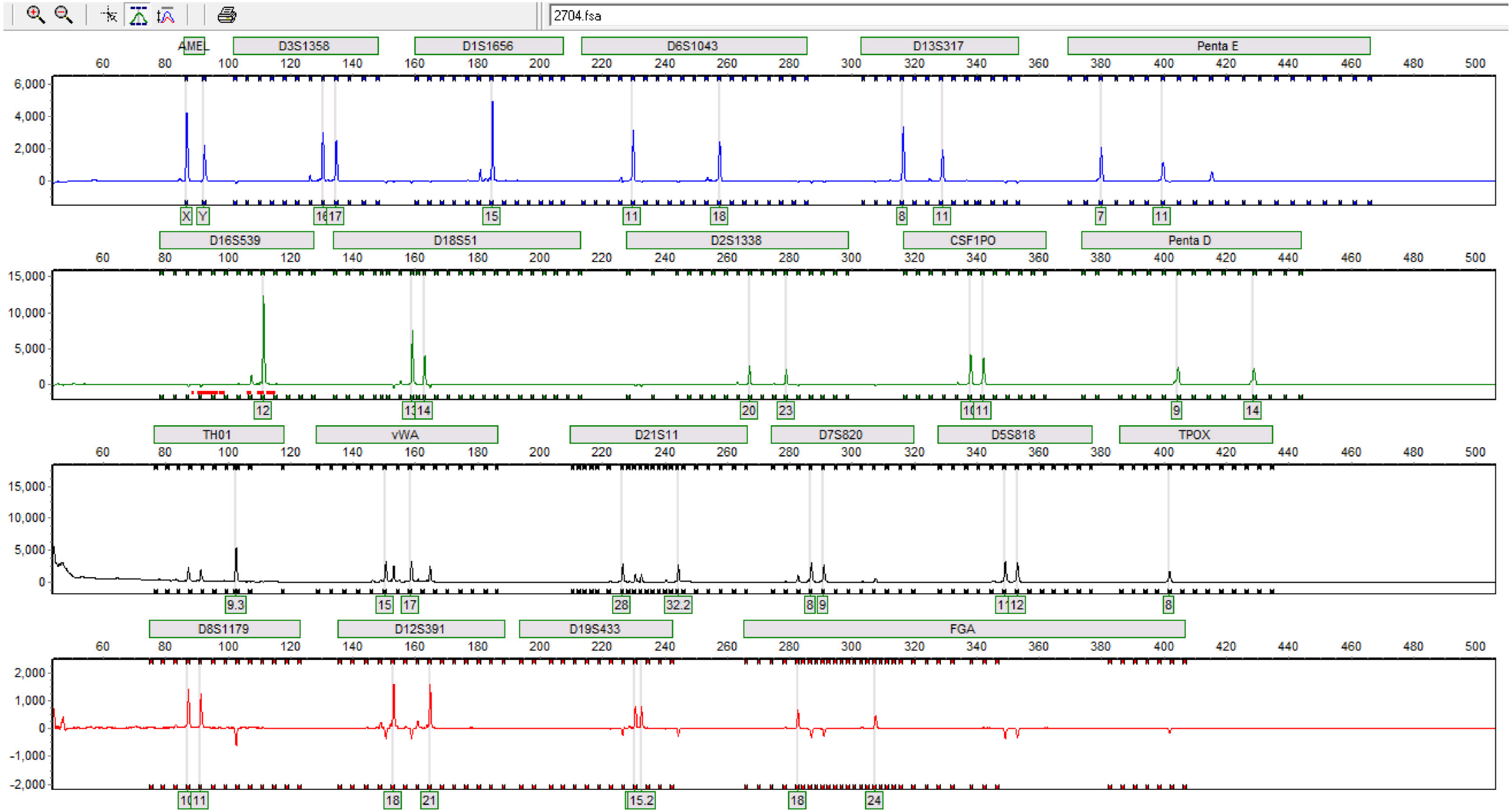

Table 1. STR profiles of U87 cell line

21	Allele1	Allele2
AMEL	X	Y
D3S1358	16	17
D1S1656	15	
D6S1043	11	18
D13S317	8	11
Penta E	7	11
D16S539	12	
D18S51	13	14
D2S1338	20	23
CSF1PO	10	11
Penta D	9	14
TH01	9.3	
vWA	15	17
D21S11	28	32.2
D7S820	8	9
D5S818	11	12
TPOX	8	
D8S1179	10	11
D12S391	18	21
D19S433	15.2	
FGA	18	24

Figure 2. Search result in ATCC database

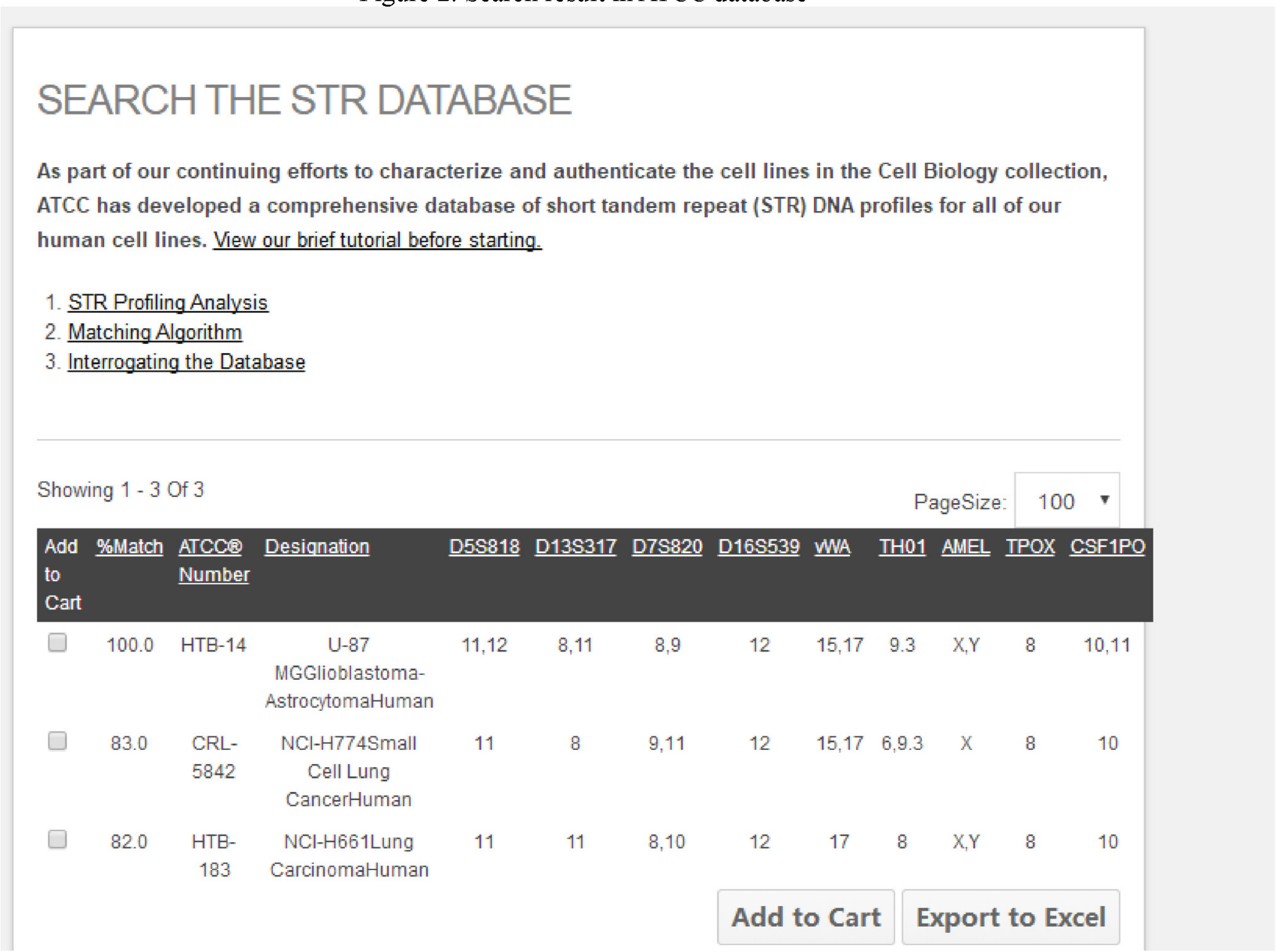

SEARCH THE STR DATABASE

As part of our continuing efforts to characterize and authenticate the cell lines in the Cell Biology collection, ATCC has developed a comprehensive database of short tandem repeat (STR) DNA profiles for all of our human cell lines. View our brief tutorial before starting.

1. STR Profiling Analysis
2. Matching Algorithm
3. Interrogating the Database

Showing 1 - 3 Of 3

PageSize: 100

Add to Cart	%Match	ATCC® Number	Designation	D5S818	D13S317	D7S820	D16S539	vWA	TH01	AMEL	TPOX	CSF1PO
	100.0	HTB-14	U-87 MGGlioblastoma-AstrocytomaHuman	11,12	8,11	8,9	12	15,17	9.3	X,Y	8	10,11
	83.0	CRL-5842	NCI-H774Small Cell Lung CancerHuman	11	8	9,11	12	15,17	6,9.3	X	8	10
	82.0	HTB-183	NCI-H661Lung CarcinomaHuman	11	11	8,10	12	17	8	X,Y	8	10

Add to Cart Export to Excel

Figure 3. Search result in DSMZ database

Result of STR matching analysis by your data.

- DSMZ Profile Database -

A graphical presentation is shown at the bottom of this page.

EV	Cell No.	Cell name	Locus names									Figures
			D5S818	D13S317	D7S820	D16S539	VWA	TH01	AM	TPOX	CSF1PO	
	Query (Your Cell)		*11,12*	*8,11*	*8,9*	*12,12*	*15,17*	*9.3,9.3*	*X,Y*	*8,8*	*10,11*	
0.94(34/36)	HTB-14	U-87MG	11,12	8,11	8,9	12,12	15,17	9.3,9.3	X,X	8,8	10,11	-

Figure 4. Authentication of the species of the sample

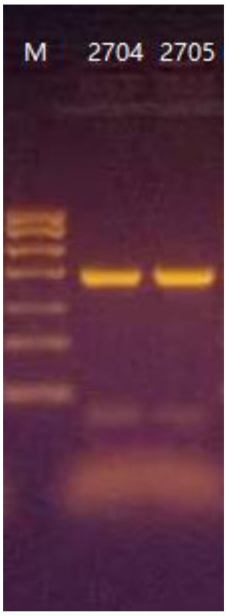

M: Marker. As the size of 700, 600, 500, 400, 300, 200 and 100bp from up to down.

Nine species are checked, as follow: *Homo sapiens* 391bp, *Cricetulus griseus*315bp, *Macaca mulatta*287bp, *Cercopithecus aethiops*222bp, *Rattus norvegicus*196bp, *Canis familiaris*172bp, *Mus musculus*150bp, *Bos Taurus*102bp, IC 70bp

JD2704: The sample. The band size is 391bp which matches the size of human.
