# Supplementary figures and images for: Expression of Concern: ZEB2 Mediates Multiple Pathways Regulating Cell Proliferation, Migration, Invasion, and Apoptosis in Glioma
Source: PLoS One. 2020 Apr 1;15(4):e0231386. doi: 10.1371/journal.pone.0231386 (PMC7112199; doi:10.1371/journal.pone.0231386)

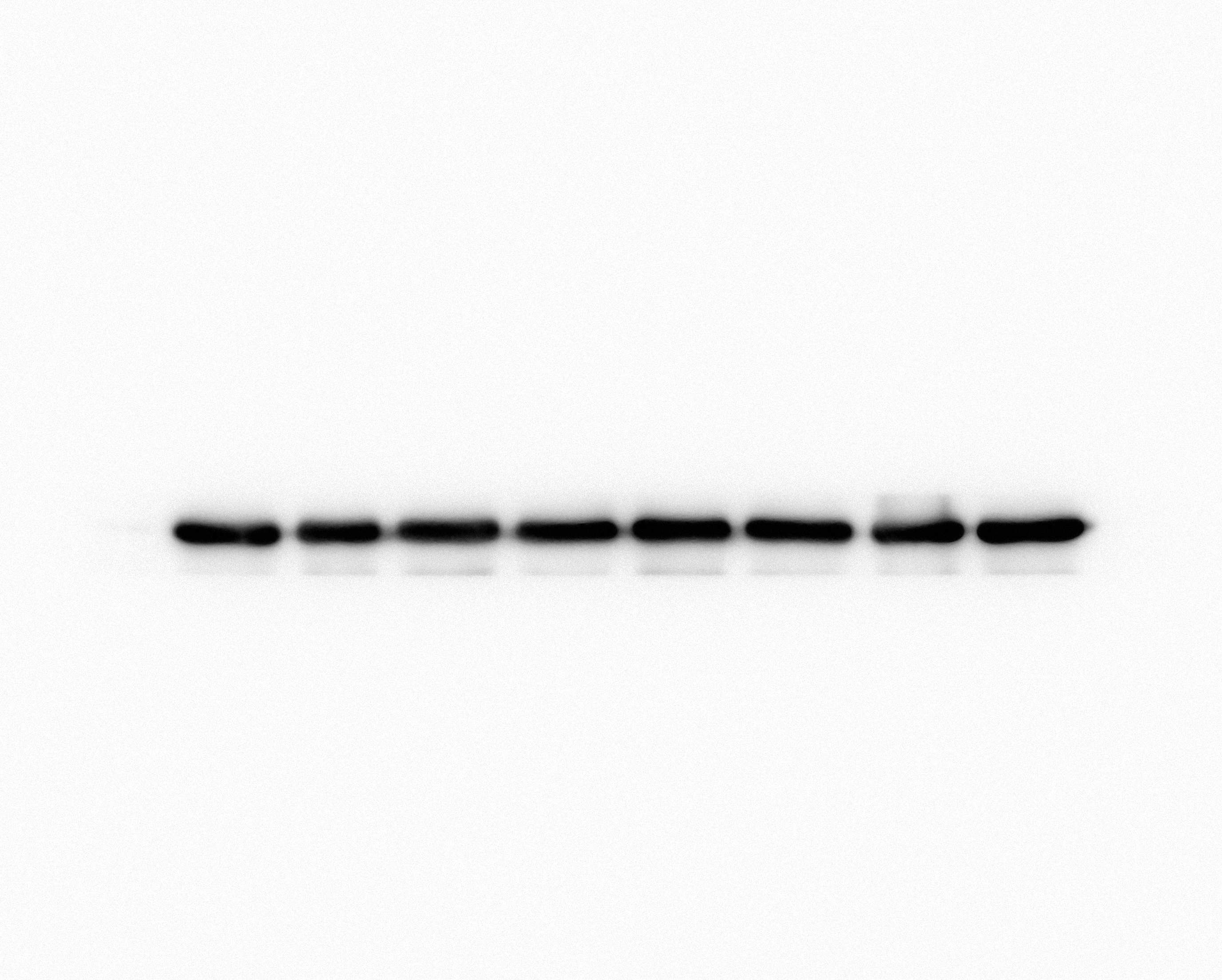

Supplement: S2 File — (ZIP) [file pone.0231386.s002.zip › File S2 Underlying western blots Fig 7/underlying blot image of actin.tif]

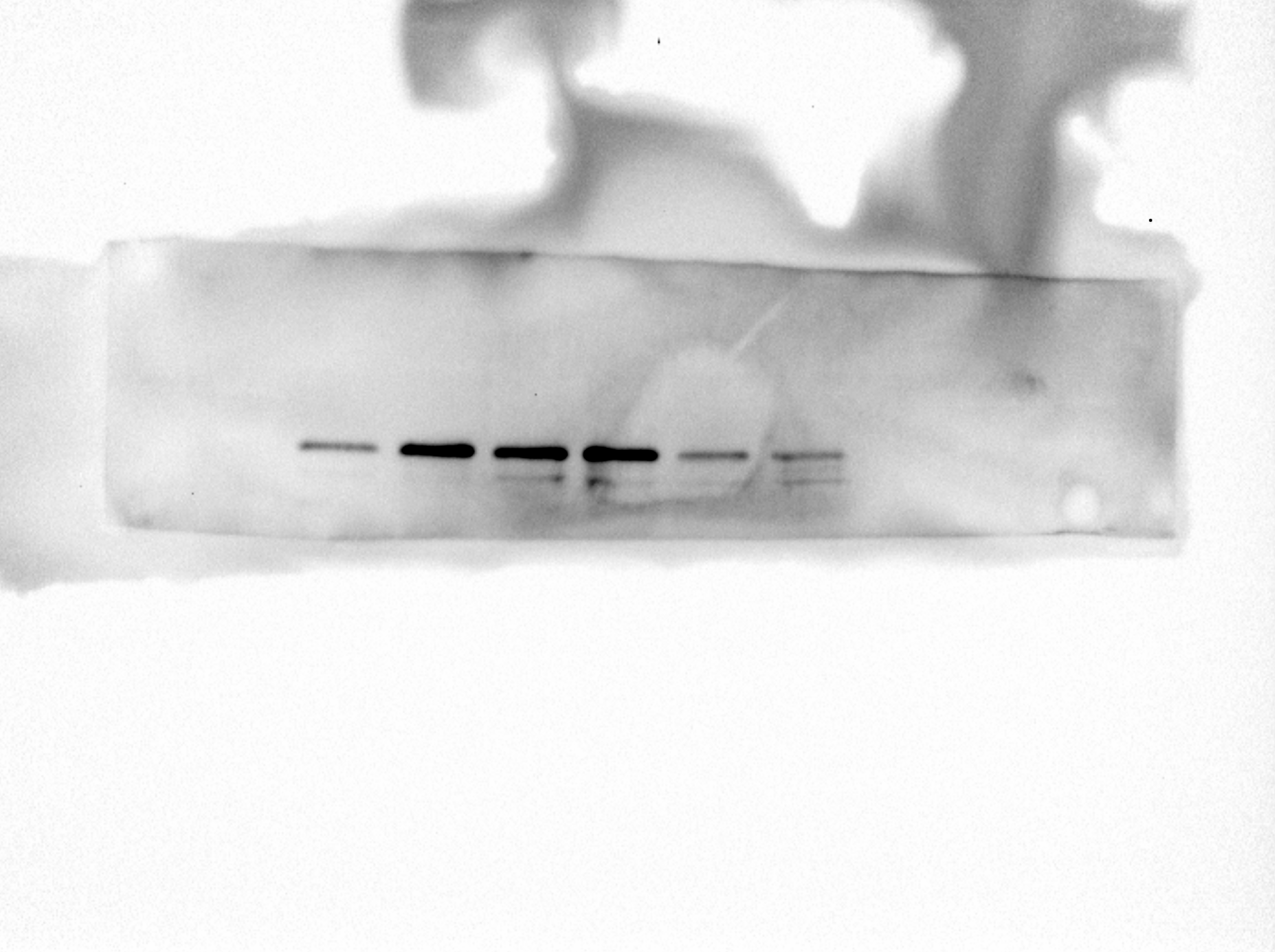

Supplement: S2 File — (ZIP) [file pone.0231386.s002.zip › File S2 Underlying western blots Fig 7/underlying blot image of capase-3.tif]

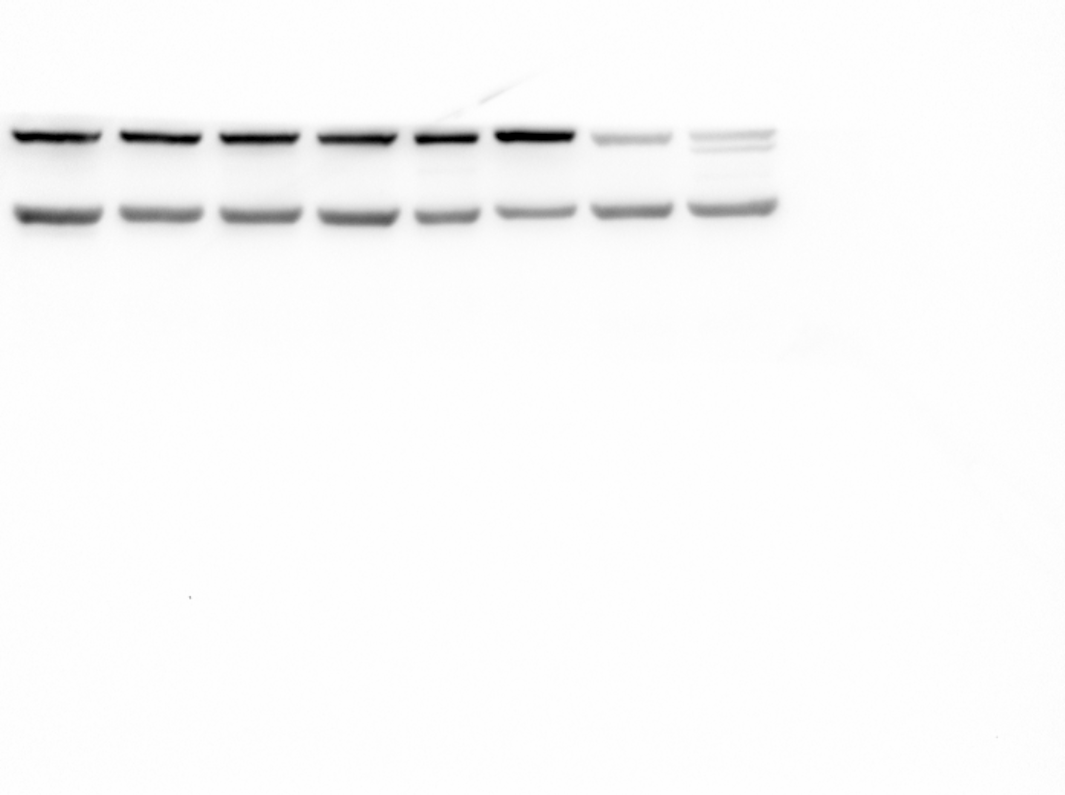

Supplement: S2 File — (ZIP) [file pone.0231386.s002.zip › File S2 Underlying western blots Fig 7/underlying blot image of capase-6.tif]

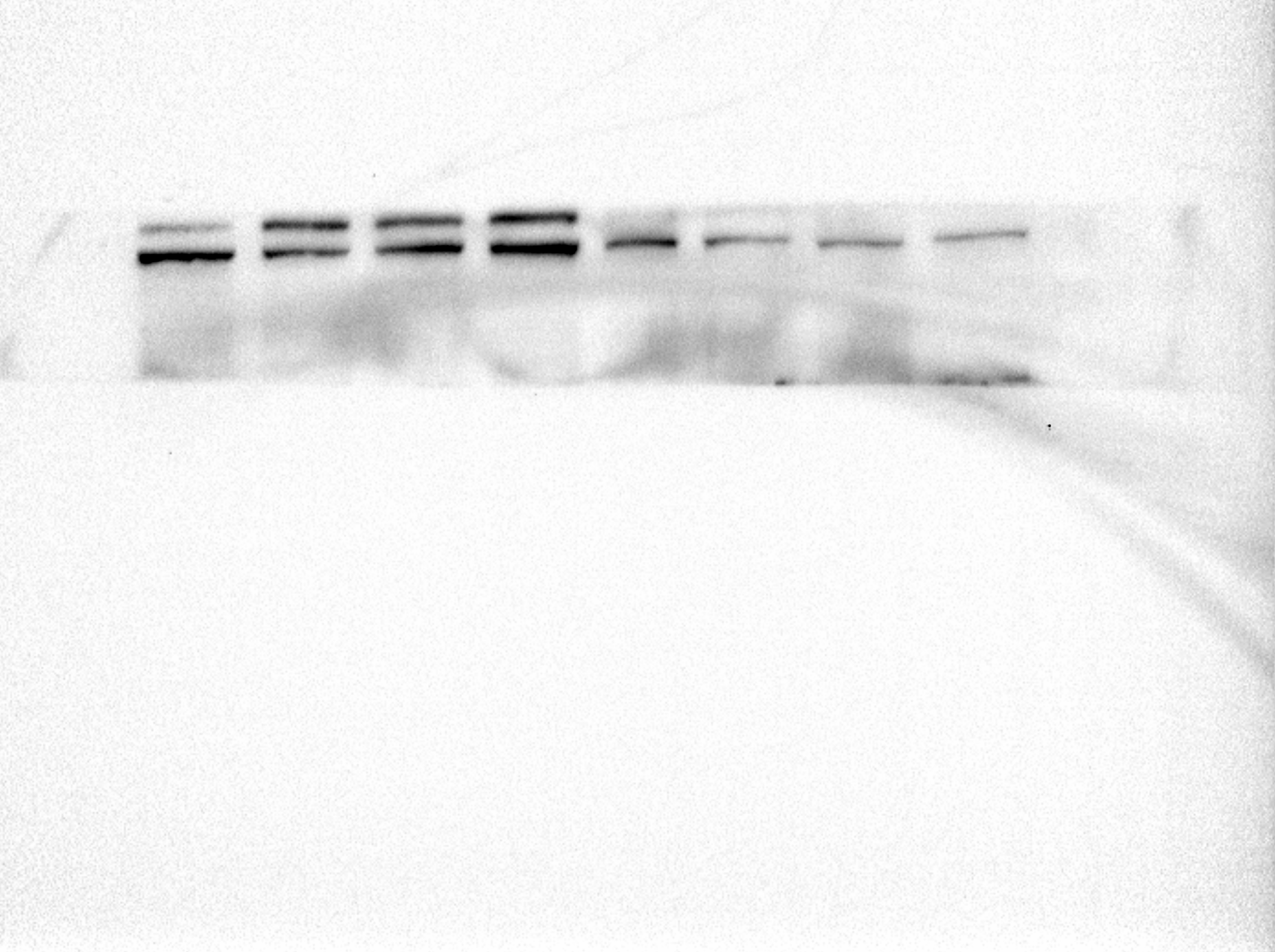

Supplement: S2 File — (ZIP) [file pone.0231386.s002.zip › File S2 Underlying western blots Fig 7/underlying blot image of capase-9.tif]

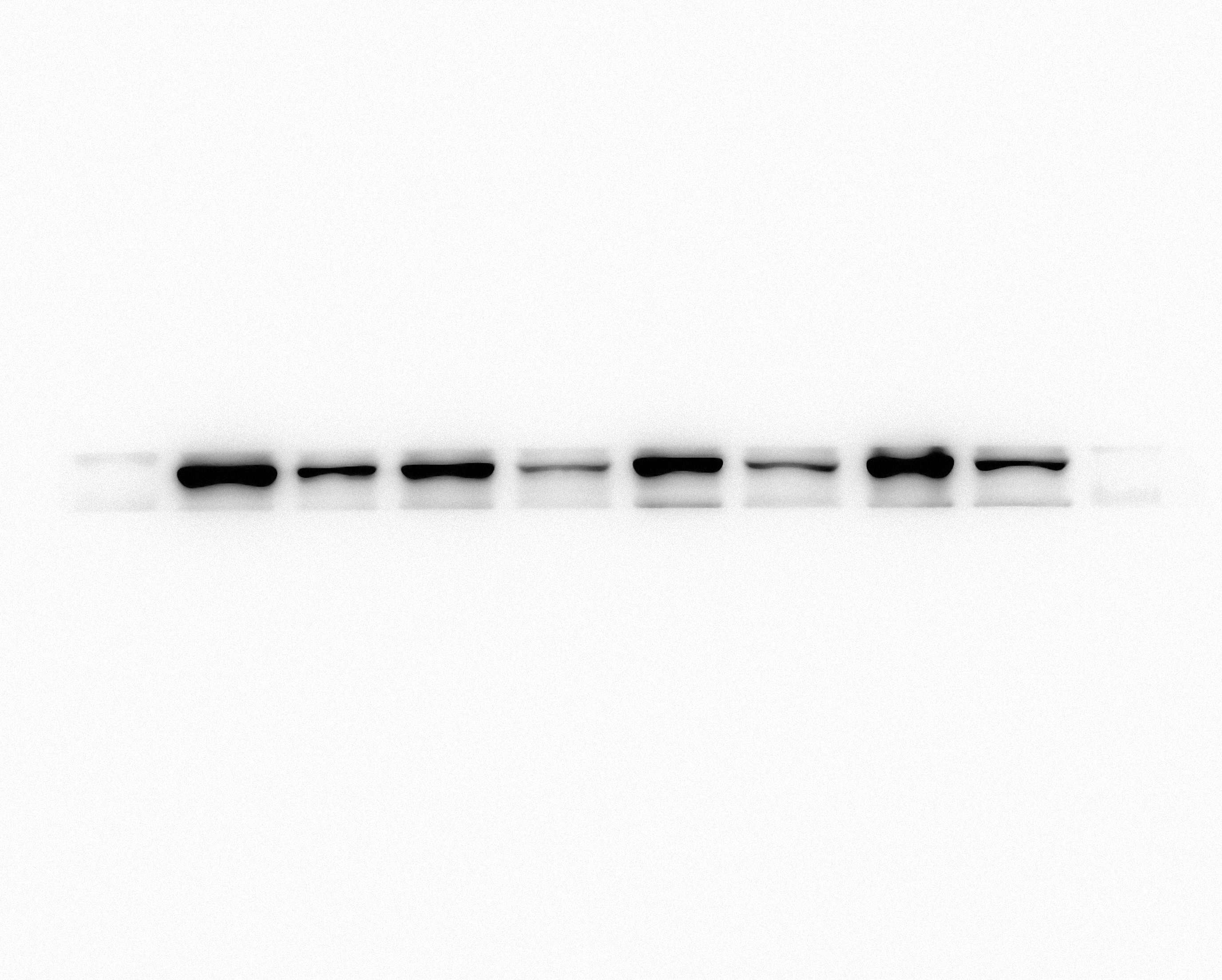

Supplement: S2 File — (ZIP) [file pone.0231386.s002.zip › File S2 Underlying western blots Fig 7/underlying blot image of Cleaved PARP.tif]

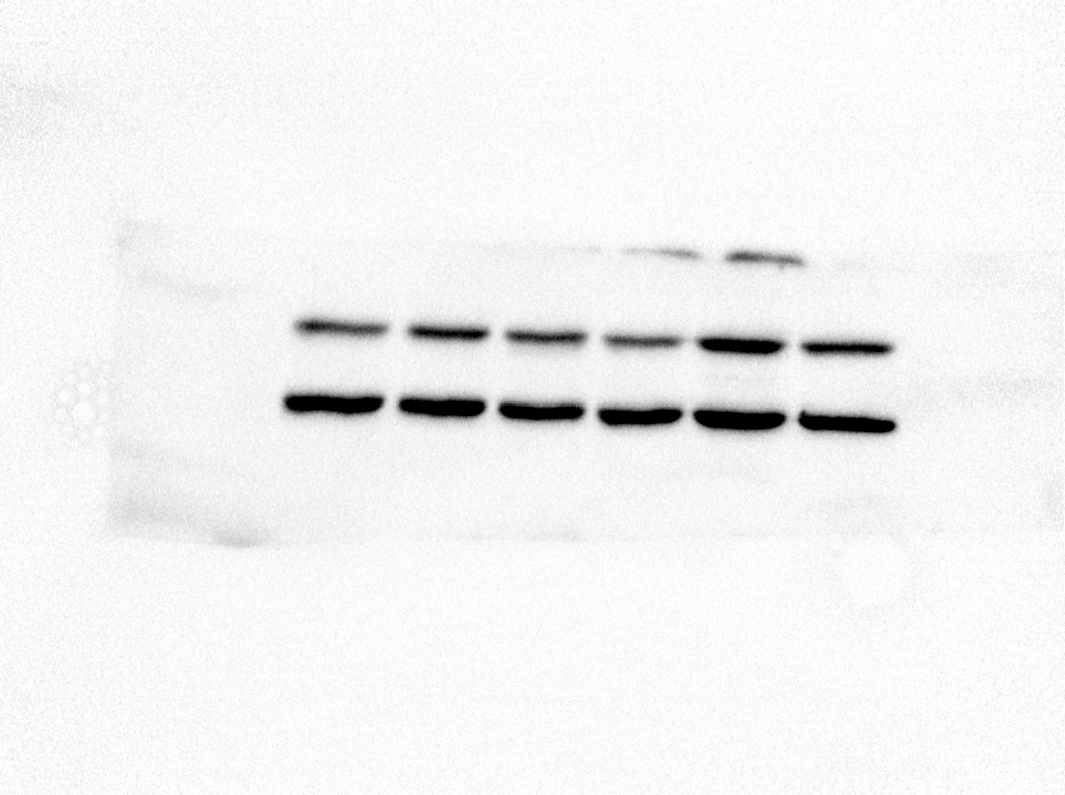

Supplement: S2 File — (ZIP) [file pone.0231386.s002.zip › File S2 Underlying western blots Fig 7/underlying blot image of PARP.tif]
