# Supplementary material for: Expression of Concern: ZEB2 Mediates Multiple Pathways Regulating Cell Proliferation, Migration, Invasion, and Apoptosis in Glioma
Source: PLoS One. 2020 Apr 1;15(4):e0231386. doi: 10.1371/journal.pone.0231386 (PMC7112199; doi:10.1371/journal.pone.0231386)
Supplement: S3 File — (ZIP) [file pone.0231386.s003.zip › File_S3/Repeat Experiment Method & Results.docx]

**Method**

***Lentivirus transfection***

Lentiviral particles carrying shRNA sequence targeting ZEB2 （Forward sequence: 5'-CCGGCTAGACTTCAATGACTATAAACTCGAGTTTATAGTCATTGAAGTCTAGTTTTTG-3'，Reverse sequence: 5'-AATTCAAAAACTAGACTTCAATGACTATAAACTCGAGTTTATAGTCATTG

AAGTCTAG-3'）or counterpart negative control sequence were constructed by GeneChem Inc. (Shanghai, China). U87 glioma cells were then transfected with these lentiviral vectors, respectively. The transfection efficacy was confirmed with WB analysis. Assays were performed in triplicate.

**Results**

**Activation of the Caspase family members were observed under shZEB2 transfection.**

In order to confirm the results we found in glioma cells with ZEB2 knockdown by siZEB2, we performed a repeated experiment with shZEB2. First, we verified that transfection of shZEB2 in U87 cells could successfully knockdown the expression level of ZEB2 (Figure 1A). Then we examined apoptosis-relate proteins mentioned in the previous manuscript. Consistent with that found in siZEB2 group, ZEB2 knockdown by shZEB2 also led to increased Caspase-3, Caspase-6 and Caspase-9(Figure 1B-D) and decreased PAPR(Figure 1E) in U87 cells, indicating induced apoptosis under ZEB2 downregulation.

**
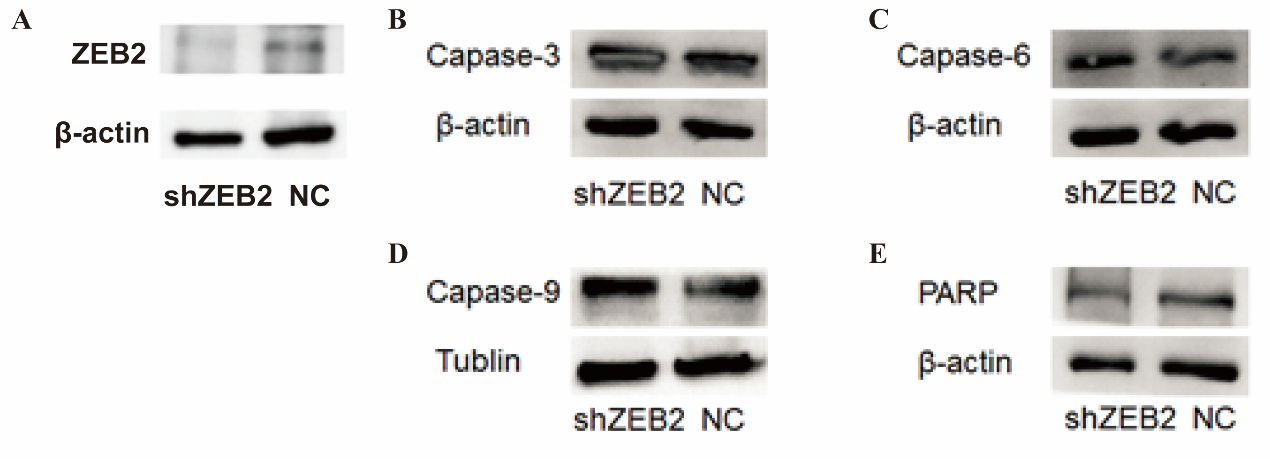
**

**Repeat Experiment Figure 1. Activation of the Caspase family members were observed under shZEB2 transfection.** Western blot was performed to confirm the expression of Caspase-3, Caspase-6, Caspase-9 and PARP in U87 glioma cells under ZEB2 knockdown. Consistently, decreased PARP and increased Caspase-3, Caspase-6 and Caspase-9 were observed after ZEB2 knockdown.
